# Analysis of (1→3) β-d-glucan as a diagnostic adjunct for invasive fungal infections in the ICU setting

**DOI:** 10.1186/cc10643

**Published:** 2012-03-20

**Authors:** N Yamada, K Shirai, T Doi, K Kumada, M Nakano, S Yoshida, I Toyoda, S Ogura

**Affiliations:** 1 Gifu University Hospital, Gifu, Japan

## Introduction

Since invasive fungal infections are associated with high morbidity and increased mortality in the ICU, early diagnosis and treatment are essential. This study assesses the performance of an assay of serum (1→3)-D-glucan (BDG) concentration in patients admitted to the ICU.

## Methods

Patients admitted to our advanced critical care center from April 2007 to March 2011 with measurements of BDG were enrolled in this retrospective study. BDG was measured when invasive fungal infection was suspected based on the Japanese guidelines for diagnosis and treatment of invasive fungal infections. BDG levels were measured using the WAKO method. A BDG level greater than 11 pg/ml was considered to be positive. No gray zone was considered.

## Results

Of the 872 patients enrolled in this study, there were 580 males and 292 females. The mean age was 60.7 years (range: 48 to 87). The mortality rate was 16.3%. We make a clinical diagnosis of invasive fungal infections according to Japanese guidelines for diagnosis and treatment of invasive fungal infections. The sensitivity of the BDG assay was 71.9% and the specificity was 91.0%. There were significant differences in sensitivity, specificity, and optimal cut-off points among patients with different clinical conditions (that is, trauma, burn, postoperative, and medical conditions).The area under the summary receiver operating characteristic curve was 0.82, but there were also differences across clinical categories.

## Conclusion

The BDG profile in ICU patients is similar to that of other inpatients. It can be useful in clinical practice if implemented in the proper setting and interpreted after consideration of the patient's clinical status.
